# Hepatitis C Virus NS3/4A Protease Inhibits Complement Activation by Cleaving Complement Component 4

**DOI:** 10.1371/journal.pone.0082094

**Published:** 2013-12-12

**Authors:** Seiichi Mawatari, Hirofumi Uto, Akio Ido, Kenji Nakashima, Tetsuro Suzuki, Shuji Kanmura, Kotaro Kumagai, Kohei Oda, Kazuaki Tabu, Tsutomu Tamai, Akihiro Moriuchi, Makoto Oketani, Yuko Shimada, Masayuki Sudoh, Ikuo Shoji, Hirohito Tsubouchi

**Affiliations:** 1 Digestive and Lifestyle Diseases, Department of Human and Environmental Sciences, Kagoshima University Graduate School of Medical and Dental Sciences, Kagoshima, Kagoshima, Japan; 2 Department of Infectious Diseases, Hamamatsu University School of Medicine, Hamamatsu, Shizuoka, Japan; 3 Miyazaki Prefectural Industrial Support Foundation, Miyazaki, Miyazaki, Japan; 4 Kamakura Research Division, Chugai Pharmaceutical, Co. Ltd., Kamakura, Kanagawa, Japan; 5 Division of Microbiology, Kobe University Graduate School of Medicine, Kobe, Japan; 6 Department of HGF Tissue Repair and Regenerative Medicine, Kagoshima University Graduate School of Medical and Dental Sciences, Kagoshima, Japan; University of Pisa, Italy

## Abstract

**Background:**

It has been hypothesized that persistent hepatitis C virus (HCV) infection is mediated in part by viral proteins that abrogate the host immune response, including the complement system, but the precise mechanisms are not well understood. We investigated whether HCV proteins are involved in the fragmentation of complement component 4 (C4), composed of subunits C4α, C4β, and C4γ, and the role of HCV proteins in complement activation.

**Methods:**

Human C4 was incubated with HCV nonstructural (NS) 3/4A protease, core, or NS5. Samples were separated by sodium dodecyl sulfate-polyacrylamide gel electrophoresis and then subjected to peptide sequencing. The activity of the classical complement pathway was examined using an erythrocyte hemolysis assay. The cleavage pattern of C4 in NS3/4A-expressing and HCV-infected cells, respectively, was also examined.

**Results:**

HCV NS3/4A protease cleaved C4γ in a concentration-dependent manner, but viral core and NS5 did not. A specific inhibitor of NS3/4A protease reduced C4γ cleavage. NS3/4A protease–mediated cleavage of C4 inhibited classical pathway activation, which was abrogated by a NS3/4A protease inhibitor. In addition, co-transfection of cells with C4 and wild-type NS3/4A, but not a catalytic-site mutant of NS3/4A, produced cleaved C4γ fragments. Such C4 processing, with a concomitant reduction in levels of full-length C4γ, was also observed in HCV-infected cells expressing C4.

**Conclusions:**

C4 is a novel cellular substrate of the HCV NS3/4A protease. Understanding disturbances in the complement system mediated by NS3/4A protease may provide new insights into the mechanisms underlying persistent HCV infection.

## Introduction

Hepatitis C virus (HCV) is a single-stranded positive-strand RNA virus of the Flaviviridae family. The viral genome encodes four structural proteins and six non-structural (NS) proteins [1]. NS3/4A, a complex consisting of NS3 with serine protease activity and cofactor NS4A, plays an essential role in processing of HCV proteins. NS3/4A is a target of direct-acting antiviral agents (DAA) [2,3], and use of an NS3/4A protease inhibitor as a DAA markedly increases the therapeutic effect of other anti-HCV agents. Thus, NS3/4A protease may play an important role in interfering with the antiviral response. 

HCV has been hypothesized to block the host immune response against persistent infection [4]. Furthermore, the time required for HCV-infected patients to develop hepatic cirrhosis varies among individuals; in particular, the progression of hepatic fibrosis seems to be slower in HCV carriers with persistent normal alanine aminotransferase (ALT) levels than in chronic hepatitis patients with elevated ALT levels [5]. These differences in clinical features might be caused by variations in the host immune response, but the underling mechanism is unclear.

In the course of proteomic analyses aimed at identifying proteins potentially involved in the pathophysiology of hepatic diseases, we found that a specific peptide fragment of complement component 4 (C4) was significantly more abundant in HCV carriers with persistent normal ALT than in patients with chronic hepatitis [6], as well as more abundant in HCV carriers, regardless of ALT levels, compared to healthy controls. Assuming that C4 expression levels are similar among these groups, this C4 fragment may be generated by post-translational processing in HCV-infected individuals. 

The complement system is part of the innate immune system, which can be activated through three pathways: the classical pathway, the mannose-binding lectin pathway, and the alternative pathway. C4, which is involved in the classical- and mannose-binding lectin pathways, can be cleaved by certain cellular protease(s), leading to a cascade of C4 activation [7]. In this study, we provide the first evidence that HCV NS3/4A cleaves C4, and that this cleavage attenuates activation of the classical pathway of complement system. 

## Materials and Methods

### Materials

HCV NS3/4A protease (217 amino acid [aa] fusion protein with NS4A co-factor fused to the N-terminus of NS3 protease domain) with His-tag, HCV core (aa 1–102) with GST-tag, and HCV NS5 (aa 2061–2302) with GST-tag were purchased from AnaSpec (Fremont, CA) or ProSpec (Rehovot, Israel). Isolated human-derived complement components (C1, C2) were obtained from Hycult Biotech (Uden, Netherlands), and C4 and C4-deficient guinea pig serum (C4d-GPS) were purchased from Sigma-Aldrich (St. Louis, MO). VX950, a HCV NS3/4A serine protease inhibitor, was obtained from Selleck Chemicals (Houston, TX). Veronal buffer, sheep erythrocytes, and hemolysin were purchased from Wako (Osaka, Japan), Nippon Biotest Laboratories Inc. (Tokyo, Japan), and Denka Seiken Co. (Tokyo, Japan), respectively.

### NS3/4A protease cleavage assay

HCV NS3/4A protease, core, or NS5 (3 μl) and 9 μl of Assay buffer (SensoLyte® 490 HCV Protease Assay Kit, AnaSpec) containing 30 mM dithiothreitol (DTT) were added to C4 (3 μl), and the mixture was incubated at 30°C for 30 min. The solution was separated by sodium dodecyl sulfate polyacrylamide gel electrophoresis (SDS-PAGE), and resolved proteins were stained with Coomassie brilliant blue (CBB). In a separate experiment, VX950 was pre-incubated with NS3/4A protease at 30°C for 30 min, and then incubated with C4 at 30°C for 30 min. Proteins detected by CBB staining were subjected to N-terminal peptide sequence analysis at Nippi Inc. (Tokyo, Japan).

### Hemolytic analysis

The method used for hemolytic analysis has been described previously [8,9]. Briefly, intermediates of complement components were sequentially added to sheep erythrocytes sensitized by hemolysin (Ab-sensitized sheep erythrocytes, EA). Dilute erythrocytes and complement components were prepared in Veronal buffer containing 2% gelatin (GVB). To prepare EA, hemolysin was added to 10 ml of erythrocytes (5×10^8^ cells/ml) and incubated at 37°C for 30 min. C1 (10 μg) was added to 5 ml of EA, incubated at 30°C for 15 min, and washed twice with GVB (EAC1). NS3/4A protease was prepared in a solution containing 20 mM Tris-HCl (pH 8.0), 20% glycerol, 100 mM KCl, 1 mM DTT, and 0.2 mM EDTA, adjusted to pH 7.5. The reaction solution was adjusted to 2 mM DTT to ensure a uniform effect on C4 activity. C4 was incubated with the NS3/4A protease, and then mixed with 100 μl of EAC1 and incubated at 30°C for 15 min (EAC1-C4). After washing twice with GVB, 1 μl of C2 (0.1 mg/ml) was added and the mixture was incubated at room temperature for 4 min (EAC1-C4-C2). After washing twice again with GVB, 150 μl of 80-fold diluted C4d-GPS was added to 30 μl of EAC1-C4-C2, and the mixture was incubated at 37°C for 30 min. The optical absorbance of the centrifuged supernatant was determined at 415 nm, and the level of hemolysis was calculated using the following formula: Hemolysis (%) = (sample OD_415_ – no C4 OD_415_)/(total hemolysis in distilled water OD_415_ – no C4 OD_415_)×100. “No C4” refers to a control sample containing EAC1 not incubated with C4. In a separate experiment, VX950 was first pre-incubated with NS3/4A protease at 30°C for 30 min, and then incubated with C4 at 30°C for 30 min.

### Cell culture and transfection

Human hepatoma-derived Huh7.5.1 cells (a kind gift from Dr. F. V. Chisari, The Scripps Research Institute, La Jolla, CA) and human embryonic kidney (HEK) 293T cells were cultured at 37°C under 5% CO_2_ in DMEM containing 10% FBS, 100 units/ml penicillin, and 100 g/ml streptomycin. DNA transfections of Huh7.5.1 cells and 293T cells were performed using Lipofectamine LTX/PLUS Reagent (Invitrogen, Carlsbad, CA) and polyethylenimine (Alfa Aesar, Heysham, Lancashire, UK), respectively. The transfection complex was formed at a DNA:reagent ratio of 1:1 (w/w) in OptiMEM (Invitrogen) with incubation for 15 min at room temperature before it was added to the culture. 

### Preparation of virus stock

The pJ6/JFH1 plasmid was generated by replacing the structural region of the JFH-1 strain with that of the J6CF strain, as described [10]. Cell culture–derived infectious HCV particles (HCVcc) were produced by introducing *in vitro* transcribed RNA from pJ6/JFH1 into Huh-7.5.1 cells by electroporation. The culture supernatant was concentrated using a 100-kDa MWCO Amicon Ultra Centrifugal Filter (Millipore, Bedford, MA). Virus infectivity was measured by indirect immunofluorescence analysis. Virus stocks (1×10^7^ focus-forming units/ml) were divided into small aliquots and stored at −80 °C until use.

### Plasmids

The C4 expression plasmid pFN21-C4A was purchased from Kazusa DNA Research Institute (Kisarazu, Japan). To create pFN21-C4A delH-Tag, the N-terminal Halo-Tag of pFN21-C4A was removed by digestion with *Hin*dIII and *Pvu*I, followed by blunt-ending with KOD FX neo (Toyobo, Osaka, Japan). pCAG-HA-NS3/4A, which expresses full-length NS3 and NS4 (derived from HCV genotype 1b, Con-1 strain) with an HA tag at the N-terminus of NS3 was generated as described [11]. Point mutation of serine to alanine at position 139 (S139A) in pCAG-HA-NS3/4A was achieved by site-directed mutagenesis using two primers: 5′-TAC TTG AAG GGC TCT GCG GGC GGT CCA CTG C-3′ and 5′-GCA GTG GAC CGC CCG CAG AGC CCT TCA AGT A-3′. The point mutation was confirmed by DNA sequencing.

### Immunoprecipitation and immunoblotting

Goat anti-human complement C4 antibody (MP Biomedicals, Santa Ana, CA) was bound to protein G–agarose beads (Thermo Scientific, Rockford, IL) in binding buffer (0.5% Nonidet P-40, 25 mM Tris [pH 7.5], 150 mM NaCl, 1 mM EDTA and protease inhibitor cocktail [Roche, Basel, Switzerland]) for 1 h at room temperature. Culture supernatants were incubated with the beads for 1 h at room temperature, and the immunoprecipitated proteins were eluted by heat treatment for 5 min at 100°C with 2× sample buffer. Culture supernatants were directly mixed with 3× sample buffer at a ratio of 1 volume supernatant to 2 volumes sample buffer (1:2 [v/v]). Cells were solubilized in lysis buffer (1% Triton X-100, 25 mM Tris, pH 7.5, 150 mM NaCl, 1 mM EDTA and protease inhibitor cocktail) on ice. Cell debris was removed by centrifugation, and the resultant supernatants were diluted 1:2 (v/v) with 3× sample buffer. Precipitated proteins, culture supernatants, and cell lysates were separated by SDS-PAGE and transferred to polyvinylidene difluoride (PVDF) membranes (Immobilon-P, Millipore). After blocking in 4% BlockAce (DS Pharma Biomedical, Osaka, Japan), the blots were incubated with the indicated primary antibodies, followed by the secondary antibody in TBST (25 mM Tris [pH 7.5], 150 mM NaCl, and 0.1% Tween 20). The primary antibodies used were anti-C4γ (clone H-291, Santa Cruz Biotechnology, Dallas, TX), anti-human complement C4, anti-HA (Sigma, St. Louis, MO), anti-HCV core (clone 2H9) and anti-GAPDH (clone 6C5, Santa Cruz Biotechnology). Donkey polyclonal Secondary Antibody to Goat IgG-H&L (HRP) (Abcam, Cambridge, UK), HRP-linked anti-mouse IgG, and HRP-linked anti–rabbit IgG (Cell Signaling Technology, Danvers, MA) were used as secondary antibodies. Finally, proteins were visualized using an enhanced chemiluminescence (ECL) reagent (ECL Select Western Blotting Detection Reagent, GE Healthcare, Little Chalfont, UK). 

### Statistical analysis

The concentration of proteins detected by Western blots was determined by densitometric analysis using the ImageJ software [12]. Statistical analysis was performed with the SPSS software (SPSS Inc., Chicago, IL) using the Tukey test, with *P* < 0.05 considered to indicate a significant difference.

## Results

### HCV NS3/4A protease cleaves C4 in vitro

**Figure 1:**
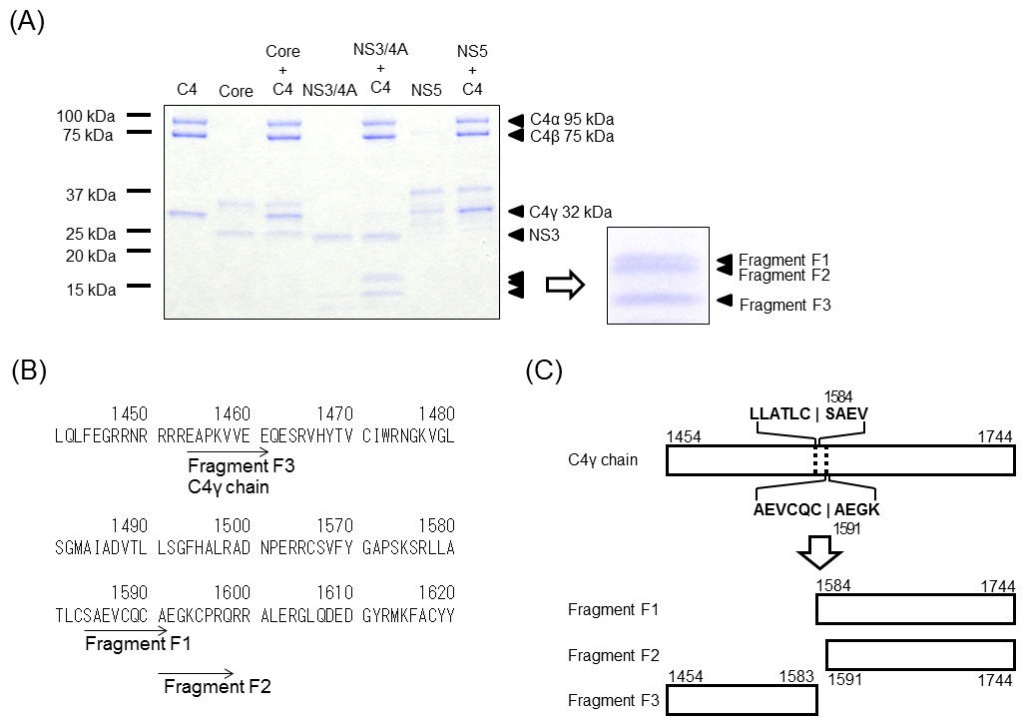
C4 is cleaved by HCV NS3/4A protease at Cys-1583/Ser-1584 or Cys-1590/Ala-1591. (A) HCV NS3/4A protease, core, or NS5 was added to C4, and the products were separated by SDS-PAGE and subjected to CBB staining. Two approximately 17-kDa proteins (Fragment F1 and F2) and a 15-kDa protein (Fragment F3) were detected after incubation of C4 with HCV NS3/4A protease, but not after incubation with core or NS5. (B) Amino acid sequence of aa 1451-1620 region of C4. Protein fragments were analyzed by N-terminal peptide sequencing. The sequences of the N-termini of the 17-kDa proteins (Fragment F1 and F2) were SAEVCQCA and AEGKCPRQ, which are located at aa 1584–1591 and 1591–1598 in C4, respectively. The sequence of the N-terminus of the 15-kDa protein (Fragment F3) was EAPKVVEE, which is located at aa 1454–1461 in C4. (C) Schematic representation of C4γ chain, and Fragment F1, F2 and F3.

To test cleavage of C4 mediated by HCV proteins, C4 (containing subunits C4α, C4β, and C4γ) was mixed with NS3/4A protease, core, or NS5, followed by incubation at 30°C for 30 min. As shown in Figure 1A, doublet bands at 17 kDa (fragments F1 and F2 in the enlarged view) and one band at 15 kDa (fragment F3) were detected in the presence of NS3/4A protease and C4. These bands were not detected after incubation of C4 with core or NS5, or when either core or NS5 were incubated alone.

N-terminal sequence analyses revealed that the bands at approximately 100, 75, and 32 kDa (Figure 1A) represented C4α (N-terminus sequence identified: NVNFQKAI), C4β (KPRLLLFS), and C4γ (EAPKVVEE), respectively. As shown in Figure 1B, the N-terminal sequences of the doublet proteins at 17 kDa were identical to sequences found in C4γ: SAEVCQCA (aa 1584–1591 of C4) and AEGKCPRQ (aa 1591–1598). In addition, the N-terminal sequence of the 15-kDa fragment was EAPKVVEE (aa 1454–1461), indicating that the 15-kDa fragment is the N-terminal region of the C4γ. These results demonstrate that HCV NS3/4A protease cleaves C4 between either Cys-1583 and Ser-1584 or Cys-1590 and Ala-1591, consistent with the consensus sequence of HCV NS3 proteinase cleavage sites [3,13]. Possible locations for the 15- and 17-kDa fragments of C4γ are shown in Figure 1B and 1C.

### HCV NS3/4A protease decreases the activity of the classical pathway of the complement system in a concentration-dependent manner

**Figure 2:**
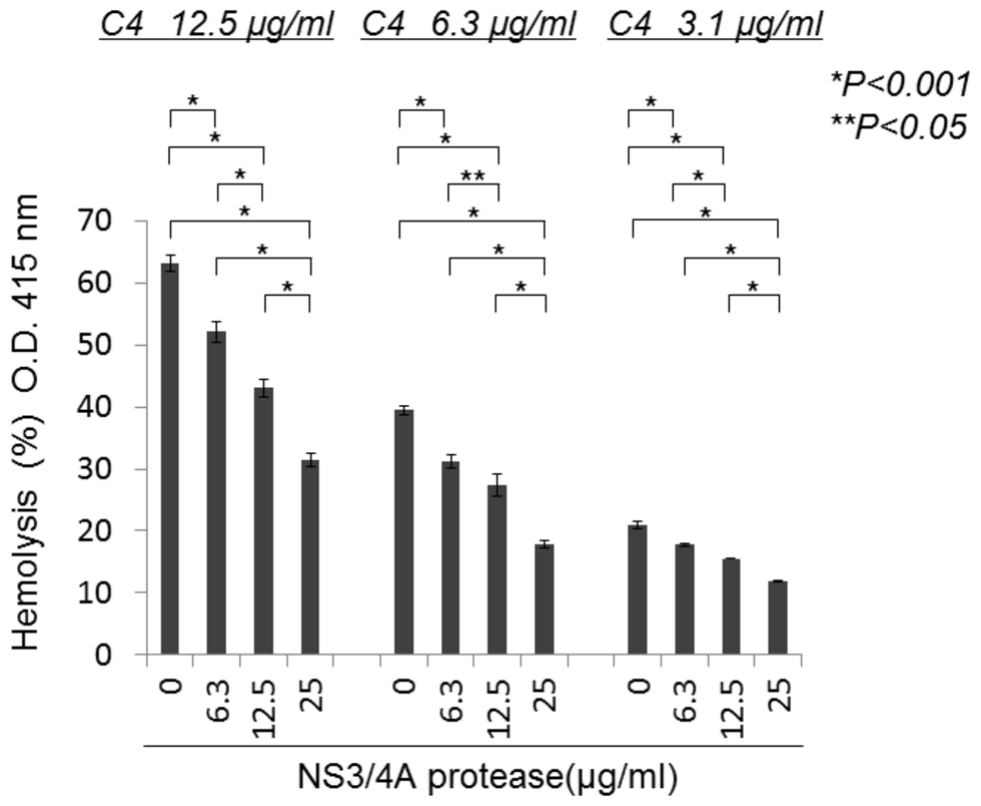
HCV NS3/4A protease inhibits the classical pathway, as assessed by hemolysis. C4 was incubated in the presence or absence of HCV NS3/4A protease, and then C1-sensitized EA (EAC1) was added (EAC1-C4). After washing, C2 was added to form EAC1-C4-C2, and the complex was resuspended in C4d-GPS. The absorbance of the centrifuged supernatant was determined at 415 nm. The grade of hemolysis decreased in the presence of NS3/4A protease in a dose-dependent manner. All measurements were performed in triplicate, and data are expressed as means ± SD.

To examine the functional significance of C4 cleavage by NS3/4A protease, complement components were serially added to EA to reproduce the classical pathway of the complement system. C4, untreated or treated with various concentrations of NS3/4A, was added at various concentrations to the EA-C1 mixture, followed by addition of C2 and C4d-GPS, which were used as sources of C3 and C5-C9. Erythrocyte hemolysis, which is caused by the complement-mediated fusion of erythrocytes, was quantified (Figure 2). NS3/4A treatment significantly decreased hemolysis levels in a concentration-dependent manner. This result, together with those in Figure 1, suggests that the C4 cleavage mediated by NS3/4A protease may contribute to inhibition of complement activation via the classical pathway.

### HCV protease inhibitor reduces inactivation of complement by blocking C4 cleavage by NS3/4A protease

**Figure 3:**
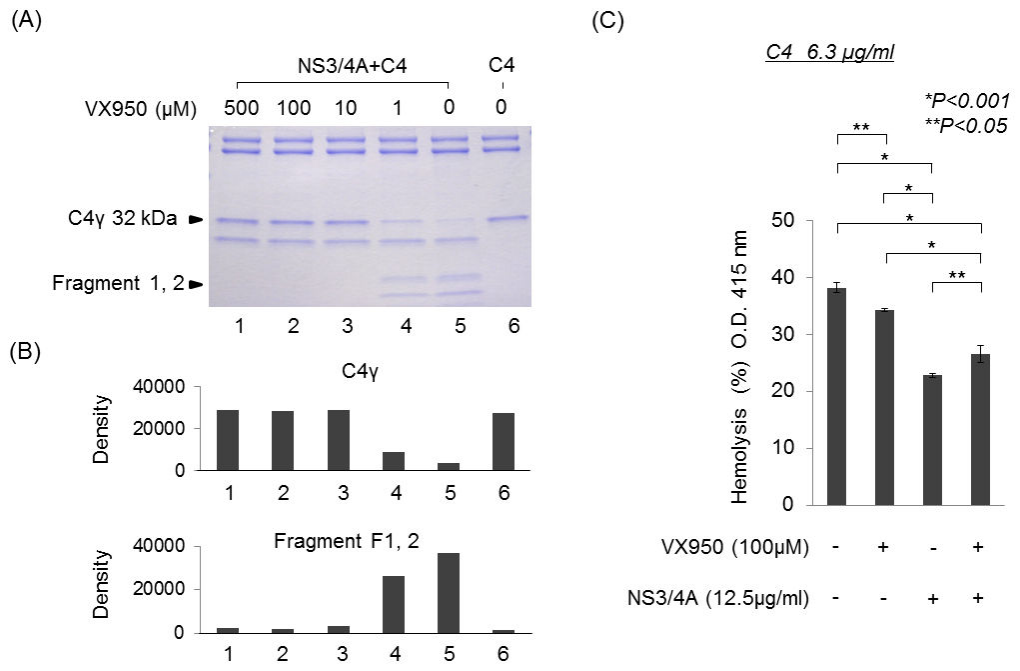
VX950, a HCV NS3/4A protease inhibitor, abrogates cleavage of C4 induced by HCV NS3/4A protease. (A) VX950 was added to HCV NS3/4A protease at the indicated concentrations, and then C4 was added. Proteins were separated by SDS-PAGE for CBB staining. The three C4-derived fragments of 17 kDa and 15 kDa produced by NS3/4A protease action could not be detected after pretreatment with VX950, and this change was accompanied by an increased concentration of the 32-kDa C4γ chain. (B) The C4γ, 17-kDa, and 15-kDa bands were quantified by densitometric analysis using the Image J software. (C) C4 was incubated in the presence or absence of HCV NS3/4A or VX950, and then C1-sensitized EA (EAC1) was added (EAC1–C4). C2 and C4d-GPS were then added, and the absorbance of the supernatant was determined at 415 nm. Hemolysis was inhibited by NS3/4A protease and this inhibition was blocked by VX950. All measurements were made in triplicate, and data are expressed as means ± SD.

We tested the effect of VX950, a specific inhibitor of NS3/4A protease, on C4 cleavage by NS3/4A protease and inhibition of complement activation. As shown in Figure 3A and 3B, under a condition in which more than 80% of 32-kDa C4γ was processed into 17- and 15-kDa fragments in the presence of NS3/4A protease (lanes 5), pretreatment of the protease with 1 μM VX950 moderately inhibited the cleavage of C4γ (lanes 4). The NS3/4A-mediated processing of C4γ into 17- and 15-kDa fragments was almost completely blocked by VX950 at ≥10 μM (lanes 1–3). In the erythrocyte hemolysis assay, the reduction in hemolysis level mediated by NS3/4A significantly recovered in the presence of VX950 (Figure 3C). These results confirmed cleavage of C4γ by NS3/4A and the involvement of the protease in the classical complement pathway. 

### Cleavage of C4γ in NS3/4A-expressing cells and HCV-infected cells

**Figure 4:**
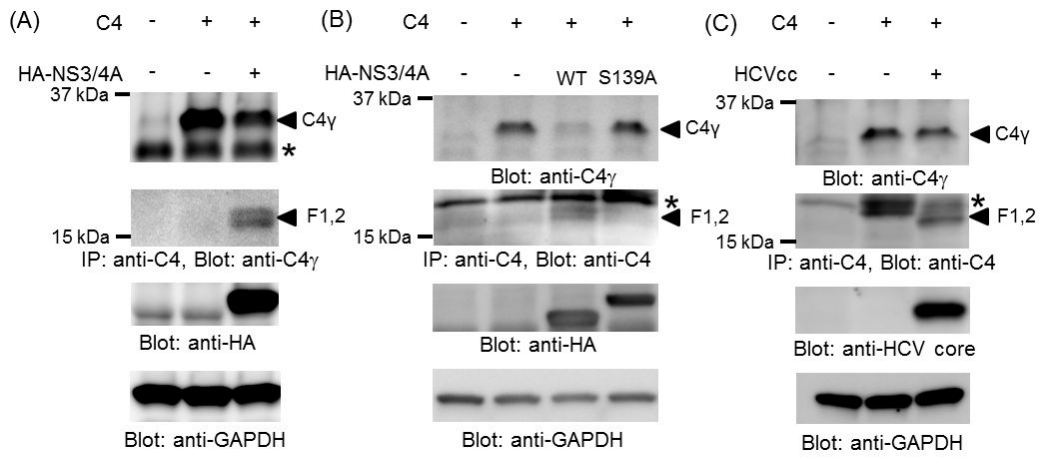
C4 is cleaved by HCV NS3/4 protease in cell cultures. (A) 293T cells were transfected with the indicated plasmids. Anti-C4 immunoprecipitates (IP) of supernatants were separated by SDS-PAGE and analyzed by immunoblotting with anti-C4γ antibody. Detergent-soluble cell lysates were separated by SDS-PAGE and analyzed by immunoblotting with anti-HA and anti-GAPDH antibodies. (B) 293T cells were transfected with the indicated plasmids. Culture supernatants were analyzed by immunoblotting with anti-C4γ antibody. Anti-C4 immunoprecipitates (IP) of supernatants were analyzed by immunoblotting with anti-C4 antibody. Detergent-soluble cell lysates were analyzed by immunoblotting with anti-HA and anti-GAPDH antibodies. (C) Huh7.5.1 cells were mock-infected or infected with HCVcc at a multiplicity of infection of 2 for 6 h, followed by mock-transfection or transfection with C4 expression plasmid. Culture supernatants and cell lysates were analyzed as described in (A) and (B). The anti-C4γ antibody was not appropriate for immunoblotting of IP samples derived from Huh7.5.1 cultures because of unavoidable nonspecific cross-reaction. * indicates non-specific reactions in (A) – (C).

To determine whether HCV NS3/4A protease cleaves C4 in cells, we analyzed 32-kDa C4γ and its processed fragments in culture medium from 293T cells cotransfected with expression plasmids encoding C4 (pFN21-C4A delH-Tag) and NS3/4A protease (pCAG-HA-NS3/4A). Co-expression of C4 and NS3/4A derived from HCV genotype 1b led to production of the 17-kDa C4γ fragment and reduction in the level of 32-kDa C4γ (Figure 4A). In contrast, the 17-kDa fragment was not detected, and the 32-kDa C4γ level was not changed, when a mutant NS3 with an amino-acid substitution at the catalytic-site (S139A)/4A was co-expressed with C4 (Figure 4B). Next, we investigated C4 cleavage in HCV-infected cultures. In the culture medium of Huh7.5.1 cells infected with HCVcc of strain J6/JFH-1 (genotype 2a) expressing of C4 from pFN21-C4A delH-Tag, the 17-kDa fragment was produced, and the level of 32-kDa C4γ was reduced accordingly (Figure 4C). These data demonstrate that C4γ can be cleaved by HCV NS3/4A, either expressed from a plasmid or in HCV-infected cells, and that proteases of both genotypes 1b and 2a are functional in this cleavage. 

## Discussion

The results of this study show that C4γ is cleaved by HCV NS3/4A protease *in vitro* and in cell culture. Cleavage of C4 by HCV NS3/4A protease leads to inhibition of activity of the classical complement pathway. C4 cleavage and abrogation of complement activation are blocked by an inhibitor of NS3/4A protease.

HCV NS3/4A protease plays an important role in the replication of non-structural regions [2,3], and might also directly act on the IFN signaling system to inhibit the host immune response and prevent viral clearance, thereby contributing to persistent HCV infection. However, a direct relationship between HCV infection and complement components has not been previously established. Levels of functional C3 or C4 assessed by hemolysis assay are reduced after infection by flaviviruses such as Dengue virus and West Nile virus (WNV) [9,14]. In mice infected with γ-herpesvirus or WNV, genetic deletion of complement C3 or C4 not only enhances mortality but also increases persistent replication of γ-herpesvirus or WNW RNA levels [14,15]. Furthermore, Moulton et al. reported that mousepox virus dissemination was more severe, and viral loads in tissues were higher, in C3-deficient mice; leading to higher mortality than in wild-type mice; those authors concluded that the complement system is critical for slowing viral spread and decreasing tissue titer and damage [16]. Thus, it is likely that the complement system is widely associated with development of viral infection. Further investigation of the role of complement activation mediated by HCV proteins such as HCV NS3/4A protease may provide new insights into development of persistent HCV infection. 

Our results indicated that the C4 cleavage site of HCV NS3/4A protease is between either Cys-1583 and Ser-1584 or Cys-1590 and Ala-1591 of C4, both of which are located in the C4γ chain (Figure 1). HCV NS3/4A protease has previously been suggested to cleave at Cys/Thr and Ala/Ser sites [3,13], which is broadly consistent with our results. C4 was also cleaved by HCV NS3/4A protease in HCV-infected cells (Figure 4C), in which unprocessed 32-kDa C4γ and cleaved 17-kDa fragment in the culture medium were observed. In cultures of human hepatoma HepG2 cells, the major fraction of C4α, C4β, and C4γ were present in the culture medium rather than in cells [17,18]. In good agreement with that finding, we detected little C4 in Huh-7–derived cells (data not shown). We speculate that immediately after synthesis, at least a fraction of C4γ can be quickly cleaved by NS3/4A in virally replicating cells, followed by secretion into the culture medium. However, we cannot rule out the possibility that HCV NS3/4A protease is present extracellularly and is functional under some particular conditions, because addition of recombinant antigens derived from the NS3 region to NS4 improves the sensitivity of the anti-HCV test in serum and shortens the window period for seroconversion in patients infected with HCV [19]. 

Complement components are involved in innate immunity and are responsible for one of the major immunological mechanisms mediated by antibodies [7]. In viral and bacterial infection, these components cause lysis of the outer membrane of virus particles [20] and infected cells [21] by the membrane attack complex C5–C9, ultimately resulting in elimination of the pathogen. Some viruses, such as cytomegalovirus, induce expression of cellular complement inhibitors, for example, decay-accelerating factor and monocyte chemoattractant protein, leading to increased levels of these proteins on the surfaces of infected cells. Human immunodeficiency virus may incorporate the complement inhibitors into the viral envelope [22,23]. NS1 protein secreted from flaviviruses, such as dengue virus, West Nile virus, and yellow fever virus, not only attenuates activation of the classical and lectin pathways by directly interacting with C4, but also inactivates C4b by interacting with C4-binding protein [9,24]. Thus, NS1 of flaviviruses is considered to play a role in protecting the virus from complement-dependent neutralization. To our knowledge, however, our study provides the first evidence that the viral protease plays a role in protecting the virus from the complement defense system via proteolytic processing of the complement component. 

In particular, C4 is involved in the classical and mannose-binding lectin pathways of the complement system, and it is responsible for the major activity of complement components. Upon antibody binding to an antigen, C4 is cleaved into C4a and C4b by the C1q-C1r-C1s complex, and C4b then binds to C2a (C4b2a) on the cell membrane to cleave C3 into C3a and C3b. Subsequently, C3b binds to C4b2a to cleave C5, and finally C5b and C6-C9 form the membrane attack complex to cause lysis of the cell membrane [7]. The erythrocyte hemolysis assay used in this study reproduces this cascade and revealed that HCV NS3/4A protease cleaves C4 and decreases the activity of the classical pathway. The specific assay was constructed to evaluate the function of C4 in the classical pathway by allowing HCV NS3/4A protease to act on C4 alone, without influence from other complement components. Therefore, further work is needed to determine whether HCV NS3/4A protease affects other components.

Several studies have demonstrated that HCV proteins influence complement systems and may be involved in evading antiviral immune responses of the host, as follows. Amet et al. reported that CD59, which may inhibit formation of the membrane attack complex, is incorporated into cultured cells and plasma primary HCV virions and inhibited activation of complement components, whereas administration of a CD59 inhibitor increases the sensitivity of component activation against endogenous HCV viral particles [25]. Banerjee et al. found that the HCV core protein reduces the expression of upstream stimulatory factor (USF)-1, a transcription factor important for basal C4 expression, and that expression of interferon regulatory factor (IRF)-1, which is important for IFN-γ–induced C4 expression, is inhibited by hepatocytes expressing HCV NS5A [26]. Mazumdar et al. showed that NS5A strongly downregulates C3 promoter activity in the presence of IL-1β, acting as an inducer [27]. HCV core inhibits T-cell proliferative responses *in vitro*, and this effect can be reversed by addition of anti–C1q receptor antibody to a T-cell proliferation assay [28]. Here, we identified C4γ as a novel cellular substrate of the HCV NS3/4A protease. 

 The results of this study suggest that C4γ cleavage by NS3/4A decreased the activity of the classical complement pathway, and might thereby attenuate activation of the complement system. An understanding of the viral protease–mediated inhibition of the complement system should provide new insights into the roles played by immune evasion in persistent HCV infection.
